# The psychology of fate: patterned constraint, situated freedom, and algorithmic prediction

**DOI:** 10.3389/fpsyg.2026.1917397

**Published:** 2026-10-16

**Authors:** Adrienne Incze, Tamás Treuer

**Affiliations:** 1 Department of Personality and Clinical Psychology, Pázmány Péter Catholic University, Budapest, Hungary; 2 Theoretical Psychoanalysis Program, University of Pécs, Pécs, Hungary; 3 Department of Psychiatry and Psychotherapy, Semmelweis University, Budapest, Hungary

**Keywords:** affordances, agency, algorithmic prediction, Daseinsanalysis, fate, predictive processing, psychoanalysis, situated freedom

## Abstract

This Conceptual Analysis rehabilitates fate as a boundary concept for theoretical and philosophical psychology. It does not treat fate as metaphysical predestination or as a new causal mechanism. Instead, fate is defined as the lived convergence of patterned constraints that organize possibility before reflective choice begins. Four features distinguish fate-like experience: temporal recurrence, pre-reflective organization of action, a felt quality of necessity, and partial rather than absolute openness to transformation. Using a transparent, targeted theory-synthesis method, the article places depth psychology in dialogue with attachment and infant research, ecological and enactive accounts of agency, predictive processing and active inference, phenomenology and Daseinsanalysis, critical and narrative psychology, and contemporary work on algorithmic mediation. The analysis clarifies that human agency is neither isolated nor exclusively internal: bodies, relationships, institutions, stories, and technological systems participate in shaping fields of action. Non-human systems may mediate and redistribute agency without thereby acquiring the same phenomenological or moral status as persons. The central construct of situated freedom names the capacity to recognize, reinterpret, resist, and transform patterned constraints by changing both one's relation to them and, where possible, the environments that sustain them. A multilevel practical heuristic is proposed for clinical, everyday, social, and algorithmic contexts. The paper also specifies limitations: fate is culturally variable, should not be used diagnostically, and must never naturalize oppression or engineered technological constraints.

## 1 Introduction: why fate belongs to future theoretical psychology

Fate sounds more at home in myth, religion, tragedy, or metaphysics than in contemporary psychology. Yet the experience it names has not disappeared. People still describe lives that repeat against conscious intention, relationships that feel uncannily familiar, and futures that seem closed before they are explicitly considered. Cross-cultural research also shows that fate is not a uniform belief: ideas of events as ‘meant to happen' vary across religious and cultural settings, and negotiable fate can preserve agency within acknowledged limits (Norenzayan and Lee, 2010; Au et al., 2012).

Psychology already offers many partial accounts of this problem. Psychoanalysis addresses repetition; attachment and developmental research implicit relational expectations; ecological and enactive approaches habits and affordances; predictive processing learned expectations and action policies; critical and narrative psychologies structural and story-shaped constraints; and research on algorithms technologically mediated choice (Bowlby, 1982; Stern et al., 1998; Clark, 2013; Segundo-Ortin, 2020; Yeung, 2017; Catena, 2026). These literatures are rich, but they rarely speak to one another.

Our question is therefore not whether psychology has concepts of agency or constraint. It does. The gap is a cross-level concept for the first-person experience in which different constraints converge into a life trajectory that feels patterned and necessary. Constraint is usually explanatory and third-person; fate names how constraint may be lived from within, as a narrowing of what can be felt, imagined, or enacted.

We use fate as a boundary concept, not as a unitary mechanism. Four criteria mark a fate-like pattern: temporal recurrence or persistence; pre-reflective organization of attention, affect, action, or possibility; a lived sense of necessity or narrowed alternatives; and partial rather than absolute openness to change. A painful event is not fate simply because it happens, nor is every social limit fate. The concept applies when constraints form a trajectory that feels already underway and difficult to interrupt.

Our central claim is simple: fate can be reformulated as patterned constraint, and freedom as situated freedom. Situated freedom is not freedom from conditions. It is the capacity to recognize, reinterpret, resist, or alter constraints by changing one's relation to them and, where possible, the environments that sustain them. This is meant to connect existing accounts of agency, relational autonomy, affordance, and social structure rather than replace them.

The argument proceeds from conceptual clarification to five explanatory domains, then to algorithmic mediation, practical use, limitations, and a research agenda.

## 2 Conceptual method: targeted theory synthesis

This is a conceptual analysis supported by a targeted narrative review. Following Jaakkola (2020) and Sukhera (2022), we make the selection logic and interpretive purpose explicit. The contribution is a theory synthesis: we bring partly separate literatures into relation in order to construct and delimit a concept, not to claim exhaustive coverage or meta-analytic integration.

Sources were selected purposively when they helped define fate or neighboring concepts, explain recurrent or pre-reflective patterning, clarify embodied, relational, social, or technological agency, or identify possibilities for change. Foundational texts were retained where historically necessary; recent work was prioritized for predictive processing, ecological-enactive agency, technology, AI, autonomy, and algorithmic ethics. We excluded uses of fate that functioned only as religious attitude, personality item, or synonym for pessimism.

We compare traditions by asking four questions: what is the main unit of analysis? What produces the pattern? How is it lived? Where can change enter? This keeps distinct mechanisms distinct. Psychoanalytic repetition, predictive priors, social inequality, and algorithmic classification are not the same process; our claim is only that they can converge phenomenologically as narrowed possibility and narratively as a life that feels compelled.

The method is selective, and the evidential status of the traditions differs. Psychoanalytic or existential concepts are not empirically confirmed by contemporary neuroscience, while computational descriptions do not exhaust the meaning of a life trajectory. Our synthesis therefore depends on preserving differences rather than translating every tradition into one vocabulary.

## 3 Conceptual architecture: fate, experience, and situated freedom

Four neighboring ideas must be kept apart. Determinism concerns causal dependence; fatalism holds that an outcome will occur regardless of action; destiny often implies calling or unfolding; and necessity here means the lived force of a pattern, not logical or causal inevitability. Fate is the more ambiguous term, spanning doom, inheritance, character, necessity, and the felt shape of a life. Bargdill (2006) notes that fate tends to emphasize constraint, whereas destiny can imply realized potential.

For this article, we define fate minimally as a cross-domain, phenomenological-structural configuration in which heterogeneous constraints become temporally self-reinforcing, organize experience and action before reflective choice, acquire the felt force of necessity, and remain only partly open to transformation. We do not posit a hidden entity or common causal mechanism behind the psychodynamic, embodied, social, or technological processes discussed below. Fate names their possible convergence in a situated life. Path dependence alone is insufficient: recurrence must also involve pre-reflective organization, lived necessity, and limited but non-zero openness to change.

This definition is historically situated. The criteria are drawn mainly from Western phenomenological, existential, and psychoanalytic traditions, placed in dialogue with contemporary psychology and cross-cultural research. Whether they travel across cultural settings is therefore an empirical question, not an assumption about experience in general.

By experience we mean the embodied, affective, temporal, and socially situated way a world becomes available. A constraint becomes fate-like when it does more than block action: it organizes what attracts attention, what solicits action, what feels credible, and what can be imagined as a future. The issue is therefore world-relation as much as inner state.

The four criteria specify this more closely. Temporal patterning means persistence, recurrence, or feedback over time. Pre-reflective organization means that perception, affect, bodily readiness, or action is shaped before explicit deliberation. Lived necessity means that alternatives feel not merely unlikely but remote, dangerous, shameful, uninhabitable, or invisible. Partial transformability distinguishes fate from strict fatalism: the pattern resists change without being absolutely immutable.

Fate, in this sense, does not compete with agency, structure, habit, affordance, or autonomy. Those concepts explain components of the pattern. Fate identifies the particular configuration in which such components become temporally organized and lived as necessity. Its contribution is synthetic and phenomenological, not mechanistic.

Situated freedom names the corresponding form of agency. We reject both sovereign voluntarism and passive fatalism. People do not choose the starting conditions of body, family, language, history, class, or technology, but they are not merely the endpoint of those conditions either. Freedom may lie in revising an interpretation, learning a new bodily response, entering a different relationship, contesting an institution, redesigning a technological environment, or acknowledging a limit that cannot be removed. The practical question is always the same: what can be changed, and at what level?

## 4 Depth-psychological sources: repetition, projection, and choice

For our argument, depth psychology matters because it shows how a pattern can be enacted before it is fully recognized. Freud's repetition compulsion describes the paradox that people may recreate painful or self-defeating situations despite conscious intentions to avoid them (Freud, 1955). What is fate-like is not recurrence alone but recurrence with the lived quality of necessity: the pattern appears intimate yet alien, enacted by the person but not fully chosen.

Jung extended the problem into symbolic and existential life. His observation that an unconscious inner situation appears outwardly as fate suggests that complexes and split-off aspects of the psyche can organize perception, attraction, avoidance, and interpretation (Jung, 1968). This need not be read as the magical claim that psyche creates external events. Its defensible clinical meaning is that an unrecognized configuration participates in shaping what is encountered as significant, threatening, attractive, or familiar, so that different situations are repeatedly lived through the same organizing pattern.

Szondi placed choice at the center of fate analysis. The unconscious, on this account, speaks not only through dreams and symptoms but through consequential selections of partners, friendships, occupations, illnesses, and modes of life (Szondi, 1952, 1963). His genetic claims and the psychometric status of the Szondi test require major qualification. The more durable point for our argument is that families transmit affective climates, identifications, prohibitions, loyalties, silences, and expectations that can shape the horizon within which choices become imaginable or livable.

Szondi's “compulsion of choice” should not be equated with the situated freedom proposed here. In Schicksalsanalyse, patterned selection is linked to familial inheritance and unconscious choice; situated freedom instead names the non-sovereign but real capacity to reflect, reinterpret, resist, and alter patterns within embodied, relational, social, and technological conditions. It is therefore a critical departure from, rather than a contemporary restatement of, Szondi's stronger hereditary and quasi-deterministic commitments: inherited patterns may shape the horizon of choice without fixing its outcome.

A contemporary reading must also avoid reducing Szondi to attachment patterns or predictive priors. Kőváry (2025) locates fate analysis within the history of existential psychology and highlights its anthropological, ethical, and existential-anancological dimensions. That broader horizon concerns how a person takes a position toward necessity, conflict, responsibility, and inherited possibility. It cannot be exhausted by a mechanistic translation. The same caution applies to analytical psychology more generally: contemporary research has investigated Jungian concepts and psychotherapy empirically, but this does not eliminate ongoing epistemological disputes about symbolic interpretation and metaphysical assumptions (Roesler, 2018, 2019).

Bollas (1989) introduces a further distinction through the idea of human idiom. Destiny can name not only compulsion but the emergence of singular form. A person may be constrained by injury but may also be drawn by an unlived direction that has not yet found expression. This complicates any simple opposition between fate and freedom: repetition can imprison, while an encounter with previously excluded possibility can feel like destiny.

The depth-psychological contribution can therefore be stated simply. What has not been integrated may organize life through repetition, projection, and choice. These traditions do not establish a universal causal law, and they must not imply that suffering, abuse, or oppression was unconsciously chosen. Their value here is to identify temporal, pre-reflective patterning and make it available for reflection.

## 5 Relational, ecological, and non-human agency

The next step is to move from intrapsychic repetition to relational patterning. Attachment theory proposes that early caregiving contributes to internal working models of self and others (Ainsworth et al., 1978; Bowlby, 1982). Mentalization theory adds that, under affective pressure, relational expectations may be enacted as certainties rather than examined as interpretations (Fonagy et al., 2002).

Stern's account of implicit relational knowing is particularly relevant because it locates patterning in procedural and affective forms of being-with, not only in explicit memory (Stern, 1985, 2004, 2010; Stern et al., 1998). A hesitation before speaking, bodily contraction during intimacy, readiness for abandonment, or attraction to emotional distance can be learned before it can be narrated. In this sense, fate may be carried as rhythm and timing: the past is repeated not because an event is consciously recalled, but because a relational form has become the default organization of encounter. What returns is therefore not only remembered content, but an embodied style of anticipating, approaching, and inhabiting the other.

Ecological psychology adds a more precise account of possibility. Gibson (1979) introduced affordances as action possibilities available in the relation between an organism and its environment. Affordances are neither simply objective properties nor subjective representations; they are relational. Chemero (2003) develops this relational interpretation, while Withagen et al. (2012) argue that affordances can invite or solicit behavior rather than merely permit it. This distinction matters for agency: a possibility may objectively exist without becoming experientially available, while another may exert a compelling bodily invitation despite its long-term costs.

For our purposes, the ecological-enactive contribution is straightforward: fate-like experience can appear as a structured and narrowed field of invitations (Gallagher, 2017). Habits and sensorimotor schemes make some affordances easier to detect and act upon than others (Gallagher, 2005), while socio-cultural norms educate attention and intention (Segundo-Ortin, 2020). A career, relationship, or public role may be formally possible yet fail to solicit action; another may feel inevitable because body and environment repeatedly stabilize it. Fate is therefore neither an inner possession nor an external force, but a recurrent way of inhabiting a world.

This also requires a more nuanced account of non-human agency. Latour (2005) argues that action is distributed across networks of human and non-human actors: doors, forms, metrics, devices, and infrastructures make differences to what can happen. Technologies do not merely stand between an already formed subject and a pre-existing world; they mediate perception and action and participate in constructing practical worlds. Postphenomenological approaches similarly show that technologies amplify, reduce, reveal, and conceal aspects of experience (Verbeek, 2011). Döbler and Bartnik (2022) extend this point by showing how technologies can create, modify, or obscure normative affordances. Contemporary posthumanist approaches likewise challenge the image of an autonomous, self-contained human subject and emphasize relational constitution across bodies, technologies, and environments (Braidotti, 2019; Ferrando, 2019). The present argument adopts this relational insight while retaining asymmetries of phenomenological experience and moral responsibility.

The claim of distributed agency should nevertheless be qualified. A recommender system or diagnostic score can have operational agency in the sense that it changes the course of action, but it does not thereby possess the same first-person experience, moral accountability, or existential responsibility as a person. Human and institutional responsibility should not disappear into an undifferentiated network. The useful conclusion is asymmetric co-agency: persons, artifacts, institutions, and environments jointly configure action, while their capacities and responsibilities remain different.

This qualification also modifies the language of a “pre-existing world.” Some material realities and historical harms precede the individual and constrain action independently of interpretation. At the same time, practical worlds are continually enacted and reorganized through relations among bodies, norms, institutions, and technologies. Fate-like constraint can therefore be inherited and manufactured, sedimented and actively reproduced.

## 6 Predictive rigidity: fate as enacted expectation

Predictive processing contributes one specific account of how recurrence can stabilize. In predictive-processing models, hierarchical systems generate expectations about sensory causes and update them in relation to prediction error (Clark, 2013; Friston, 2010). Active inference extends this framework to action and policy selection (Parr et al., 2022; Pezzulo et al., 2024). We use this as one explanatory route, not as a master theory of fate.

The language of prior beliefs is easily oversimplified. Priors differ in precision, and precision determines how strongly a prediction is weighted relative to incoming evidence. Habits can also be modeled as prior beliefs over policies: some courses of action become likely because they have repeatedly reduced uncertainty or threat. A fate-like pattern can therefore be described, cautiously, as a configuration in which high-precision expectations and habitual policies restrict exploration. The person does not merely predict rejection; attention, bodily readiness, interpretation, and action may be organized so that ambiguous situations are sampled as threatening and protective routines are repeatedly selected.

This account is not committed to a purely internalist picture. Ecological and enactive interpretations of active inference emphasize action-oriented organism-environment coupling rather than a detached brain constructing an internal replica of the world (Bruineberg et al., 2018; Ramstead et al., 2020). This debate is important because fate as patterned constraint concerns both learned expectations and the environments that stabilize them. A person may anticipate danger because of an outdated model, because the present environment is genuinely unsafe, or because both processes interact.

Predictive processing therefore does not prove Freud, Jung, or Szondi; the concepts operate at different explanatory levels. Its narrower contribution is to specify one route by which recurrence acquires stability: precision-weighted expectations guide sampling, and sampling can confirm expectations. The resulting feedback loop can make a contingent model feel like a necessary world.

The framework also clarifies change. New information is not sufficient if it is dismissed as unreliable or if the person lacks safe opportunities for exploration. Corrective experiences must be salient, repeated, embodied, and socially credible enough to alter not only beliefs, but the bodily and relational sense of what can be safely tried. Psychotherapy can sometimes provide such conditions: expected rejection is met with recognition, expected humiliation with curiosity, or expected domination with negotiated difference. Yet no individual can update a model of safety while remaining in persistently unsafe conditions. Predictive explanations must therefore remain integrated with social and material analysis.

A further limitation is that predictive-processing language can create an illusion of technical certainty. Current theories disagree about representation, inference, embodiment, and the explanatory reach of the free-energy principle. The present article uses predictive rigidity as a constrained interpretive proposal, not as an established biomarker or a universal computational diagnosis of fate.

## 7 World-openness, social constraint, and narrative

Phenomenology changes the question. Instead of asking only what mechanism repeats, it asks what kind of world is disclosed and which possibilities can appear within it. Drawing on Heidegger, Binswanger and Boss understood psychological suffering as a disturbance of being-in-the-world rather than merely an event inside a psychic container (Binswanger, 1963; Boss, 1963; Heidegger, 1962).

This approach deepens the meaning of experience. Depression may involve not simply negative beliefs but a future that has lost openness; anxiety may involve a world that solicits vigilance; trauma may involve a present in which danger is not remembered as past but encountered as imminent. Condrau's continuation of Daseinsanalysis allows familial inheritance to be understood as inherited worldhood: families transmit not only stories or prohibitions, but tacit orientations toward what can be spoken, what must remain silent, what counts as shameful, what love requires, and what freedom threatens (Condrau, 1998). Fate is then a narrowed horizon of possibility, not only an intrapsychic content.

Rollo May's account of freedom and destiny offers an existential bridge. Freedom is exercised within body, history, mortality, culture, and responsibility rather than outside them (May, 1981). This resonates with relational approaches to autonomy, which reject the fiction of an isolated chooser and emphasize that self-governance depends on social recognition, competencies, and available options (Mackenzie and Stoljar, 2000). Situated freedom extends this insight by adding temporal patterning and lived necessity: the issue is not only whether options exist, but whether they can appear as inhabitable possibilities.

Critical psychology adds an indispensable ethical correction. Poverty, racism, gendered violence, class immobility, war, migration, and institutional exclusion are not projections or failed updates. They are real constraints that shape bodies, relationships, and futures (Adams et al., 2015; Parker, 2007). The language of fate becomes harmful when it psychologizes oppression or shifts responsibility from institutions to individuals. Situated freedom therefore includes collective and political action, not only intrapsychic insight.

Cultural and narrative psychologies further complicate universality. Fate attributions vary across cultural and religious settings, and negotiable fate can function as a way of sustaining agency under conditions that cannot be controlled (Norenzayan and Lee, 2010; Au et al., 2012). Narrative identity organizes reconstructed past, lived present, and anticipated future into a provisional whole (Bruner, 1990; McAdams, 1993). A family secret, repeated loss, or chance meeting can acquire the meaning of destiny retrospectively. Narrative can disclose a pattern, but it can also imprison the person in a closed identity when it turns a historically formed way of finding oneself in the world into an apparently essential self.

Art remains relevant not as evidence but as phenomenological disclosure. Tragedy makes visible how action, blindness, inheritance, and recognition can become entangled: attempts to escape a feared outcome can participate in bringing it about. It shows, in compressed form, how a life can be both authored and inherited, constrained and responsible.

## 8 Algorithmic fate: sociotechnical prediction and the manufacture of possibility

Algorithmic systems are our contemporary test case because prediction can become part of the environment it predicts. Ranking, recommendation, classification, allocation, and automation can alter visibility, access, attention, opportunity, and the lived accessibility of future options. Research on digital autonomy distinguishes mediation, in which systems shape deliberation and behavior, from automation, in which systems perform or constrain decisions that would otherwise involve human judgment (Catena, 2026; Rouvroy and Berns, 2013).

Algorithmic fate should be reserved for sociotechnical configurations with four features: prediction is used to organize consequential action; the resulting classification creates feedback that affects future options; the process is opaque or difficult to contest; and the effects persist across time or domains. A recommendation that merely suggests a film is not fate. A risk score that repeatedly affects access to health care, insurance, employment, credit, or social support is closer to the concept because the prediction participates in producing the trajectory it claims to forecast.

Several mechanisms are relevant. Recommender systems curate attention and can gradually shape preference environments (Milano et al., 2020). Hypernudges use real-time data and adaptive choice architecture to influence conduct at scale (Yeung, 2017). Manipulative systems can exploit vulnerabilities while preserving the appearance of voluntary choice (Susser et al., 2019). Algorithmic abstraction can also detach technical categories from the social contexts in which fairness, discrimination, and due process acquire meaning (Selbst et al., 2019). These are not metaphysical forces; they are engineered arrangements with owners, designers, incentives, and governance structures.

The consequences are not hypothetical. Obermeyer et al. (2019) showed that a widely used health-management algorithm underestimated the needs of Black patients because health cost was used as a proxy for illness. The example illustrates performative prediction: historical inequality enters a proxy, the proxy structures allocation, and the allocation can reproduce inequality. Relational approaches to algorithmic injustice emphasize that harm emerges within networks of power and social dependence rather than from isolated technical errors alone (Birhane, 2021).

AI systems can also appear agentic and authoritative, which may encourage overreliance, automation bias, deskilling, and the delegation of judgment. Recent analyses describe risks to understanding, decision-making competence, voluntariness, authenticity, and relational autonomy, as well as forms of algorithmic paternalism (Hofmann, 2026). The important distinction is between operational and moral agency. Algorithms can select actions and reorganize environments, but responsibility remains distributed among developers, institutions, deployers, regulators, and users. Meaningful human control requires that decisions remain traceable to reasons, responsive to relevant human values, and contestable by those affected (Santoni de Sio and van den Hoven, 2018).

Technological mediation also changes affordances. Interfaces make some actions easy and salient while hiding others; enhancement technologies can create new capacities while establishing new norms of expected performance (Döbler and Bartnik, 2022). This is where the transhuman promise of overcoming limitation intersects with fate (Bostrom, 2005). Technology may expand agency, but optimization can also narrow the range of acceptable bodies, emotions, tempos, and life courses. A culture that treats every vulnerability as a defect risks converting contingent standards of performance into moral necessities.

The purpose of the term algorithmic fate is therefore critical, not mystifying. It should reveal how predictions become environments and how environments feed back into persons, without naturalizing technological constraints as inevitable. The relevant interventions are practical: transparency about proxies, bias audits, intelligible explanation, accessible appeal, alternative pathways, limits on manipulation, and institutional accountability. Algorithmic fate is manufactured; it can therefore be redesigned and contested.

## 9 From concept to practice: a multilevel heuristic

The framework is useful only if it helps locate intervention. Table 1 translates the argument into five domains rather than labeling a person, group, or trajectory as simply fated. The domains can interact: an expectation of abandonment may be embodied as vigilance, narrated as personal defect, reinforced by an exploitative relationship, and amplified by a digital environment that repeatedly recommends confirming content.

**Table 1:** Multilevel heuristic for fate as patterned constraint.

Domain	Patterning process	Lived or practical form	Transformative leverage	Ethical caution
Unconscious and relational	Repetition, projection, attachment expectations, implicit relational knowing	‘I keep entering the same relationship'; conflict feels familiar and inevitable	Interpretation, mentalization, corrective relationship	Do not imply that abuse or trauma was chosen.
Embodied and ecological	Habits, sensorimotor schemes, selective salience, narrowed affordance field	An available option does not feel inhabitable; the body contracts before deliberation begins	Embodied practice, graded exploration, environmental redesign	Avoid treating formal opportunity as lived accessibility.
Predictive and active-inferential	High-precision expectations and habitual policies restrict exploration and bias sampling toward confirming evidence.	Safety feels unsafe; ambiguous cues are read as rejection or danger	Repeated, credible disconfirming experiences; alternative policies; secure conditions	Do not reduce meaning to neural computation.
Social, cultural, and narrative	Institutions, norms, material inequality, family and cultural narratives	‘People like me do not get another future'; roles and opportunities are structurally narrowed	Collective action, material support, narrative reconfiguration, policy change	Do not psychologize oppression.
Algorithmic and sociotechnical	Profiling, ranking, recommendation, proxy variables, automated allocation, feedback loops	A score or classification repeatedly shapes visibility, access, and future data	Audit, explanation, appeal, alternative pathways, meaningful human control	Do not naturalize engineered systems as destiny.

In clinical work, the same recurrent withdrawal from intimacy can be read at several levels: defensive repetition, expectations of unavailability, a failure of safety cues to solicit approach, high-precision threat expectations, or genuinely unsafe relationships. The point is not to accumulate explanations, but to identify which level is active and where change is possible.

Outside therapy, the relevant level may be institutional or technical. An applicant repeatedly classified as high risk by automated systems does not primarily need insight into an unconscious script; the questions concern data, proxies, rules, and appeal mechanisms. Situated freedom may therefore require interpretation in one case and political or technical contestation in another.

## 10 Objections, limitations, and ethical safeguards

The framework should also survive its strongest objections. First, situated freedom is not new in the broad sense: existential, phenomenological, feminist, and critical traditions have long argued that freedom is exercised within conditions. Our narrower contribution is to specify fate-like experience through four criteria and connect explanatory levels without treating them as equivalent.

A stronger objection is that “fate” adds nothing to path-dependent, self-reinforcing constraint. We accept this as a limiting test. Where third-person descriptions of constraint are sufficient, the term should be omitted. Its additional work is restricted to cases in which patterned constraint is also lived as an already unfolding trajectory that narrows what can credibly be imagined or inhabited.

A second concern is overbreadth. If every constraint is called fate, the concept becomes empty. The proposed criteria are meant to prevent this. The term should be abandoned whenever a more specific explanation is sufficient or when fate obscures rather than clarifies.

A third concern is universality. The framework does not assume that all people experience constraint as fate or that its domains are culturally invariant. The criteria arise from a predominantly Western intellectual genealogy, and meanings of agency, selfhood, responsibility, and destiny vary across traditions. We therefore offer a heuristic for a family of experiences, not an ontology of every life. Empirical and qualitative research must test how the concept travels across settings.

A fourth concern is ethical misuse. In clinical contexts, fate language can imply victim-blaming, especially when trauma, violence, or structural exclusion are involved. We therefore separate participation in a pattern from responsibility for its origin: a person may enact a protective adaptation without having chosen the conditions that made it necessary. The task is to expand possibility, not assign blame.

A fifth concern is technological mystification. Calling an algorithmic outcome fate must not make engineered systems appear natural or inevitable. Analysis should identify the responsible organization, the data and proxy structure, the options affected, and the available routes of contestation.

Finally, the evidential basis is heterogeneous. We synthesize historical theory, empirical psychology, philosophy, and technology studies without claiming a single empirical model. The framework is therefore a proposal for recognizing lived patterned constraint across explanatory domains; its criteria and heuristic still require operationalization and testing.

## 11 Research agenda

The research agenda follows directly from what remains unresolved. First, qualitative and phenomenological studies should examine how people distinguish bad luck, structural exclusion, habit, compulsion, destiny, and fate, and how those distinctions vary across cultures.

Second, measurement research should distinguish fate-like experience from fatalism, external locus of control, hopelessness, and low self-efficacy. The four criteria are unlikely to be equally measurable: recurrence can be studied longitudinally and perceived transformability through self-report or behavioral designs, whereas pre-reflective organization may require process-sensitive methods. Felt necessity is likely to resist simple psychometrics and may be better studied through phenomenological interviews or mixed methods.

Third, longitudinal studies could investigate whether relational expectations, affordance perception, habitual policies, and narrative identity form feedback loops that predict persistent trajectories. Experimental and clinical work might examine whether changes in bodily skill, relational safety, environmental opportunity, or narrative complexity broaden perceived affordances and reduce the felt necessity of a pattern.

Fourth, research on algorithmic systems should ask when predictions become self-reinforcing. Key variables include opacity, duration, domain spillover, proxy bias, access to appeal, and adaptation to anticipated classification. Participatory methods are important because affected people may see constraints that designers do not.

Finally, psychotherapy research should examine whether fate language helps particular patients recognize patterns or instead increases passivity and blame. Its clinical use should be tested as an intervention in meaning and lived possibility, not assumed to be beneficial.

## 12 Conclusion

Fate has not disappeared from modern life; it has been redistributed across psychological, social, and technological vocabularies. The processes reviewed here are not interchangeable mechanisms. Their relevance to fate lies in the possibility that they converge in a lived trajectory that feels already underway, narrows what can be imagined or inhabited, and yet remains not wholly closed.

Used in this limited sense, fate is neither predestination nor diagnosis. It is a boundary concept for the point at which patterned constraint becomes a world. Situated freedom names the corresponding task: not sovereign escape from conditions, but the situated capacity to reinterpret, embody differently, revise relationships, contest institutions, redesign technologies, or acknowledge limits without confusing them with necessity.

For theoretical psychology, the value of the concept is connective and critical. It links first-person experience with relational, social, and sociotechnical conditions while preserving differences of mechanism and responsibility. Algorithmic prediction makes this especially visible because engineered classifications can reorganize access and possibility without becoming moral agents. The test of the concept is therefore pragmatic as well as empirical: it is useful only where it reveals how apparent inevitability is produced, where change might enter, and where responsibility properly lies.
